# Control of the *Pseudomonas aeruginosa* LbcA•CtpA proteolytic complex and its substrates

**DOI:** 10.1128/jb.00169-25

**Published:** 2025-10-08

**Authors:** Kévin J. Rome, Andrew J. Darwin

**Affiliations:** 1 Department of Microbiology, NYU Grossman School of Medicine 171480, New York, New York, USA; Geisel School of Medicine at Dartmouth, Hanover, New Hampshire, USA

**Keywords:** cell envelope, regulation, protease, *Pseudomonas aeruginosa*

## Abstract

*Pseudomonas aeruginosa* is a highly adaptable bacterial pathogen with a resilient cell envelope. This envelope must be elongated as cells grow, which requires coordinated biosynthesis of the inner and outer membranes and the peptidoglycan cell wall. Cell wall endopeptidases are essential to expand the peptidoglycan sacculus, and the LbcA•CtpA proteolytic complex controls the activity of multiple endopeptidases by degrading them. Here, we report an investigation into control of the LbcA•CtpA proteolytic complex and its substrates. LbcA and CtpA levels were unaffected by growth rate, which corresponded with constitutive expression of their genes. For CtpA, this was explained by its arrangement in a complex operon containing an internal *ctpA* promoter. Despite constitutive LbcA and CtpA production, the LbcA•CtpA substrate levels were higher when cells were growing rapidly. In most cases, this correlated with modestly higher substrate gene expression in the exponential phase. However, most of the control came from reduced CtpA activity when cells were growing rapidly. Our data suggest that CtpA activity might be affected by phospholipid transport and related processes in the cell envelope. A similar phenomenon was reported to affect the *Escherichia coli* NlpI•Prc complex, even though there are major sequence and structural differences between the NlpI•Prc and LbcA•CtpA complexes. This makes it likely that growth-rate-dependent autolysin control by these proteolytic complexes is widely conserved, even if they are composed of non-orthologous proteins in some cases.

**IMPORTANCE:**

Carboxyl-terminal processing proteases occur in all domains of life. Some are associated with bacterial virulence, including *P. aeruginosa* CtpA, which works with the outer membrane lipoprotein LbcA to degrade cell wall endopeptidases. We report that the LbcA•CtpA complex activity is coordinated with growth rate, ensuring appropriate levels of its substrates for cell wall expansion. The mechanism appears to be connected to phospholipid transport, much like a phenomenon reported for *Escherichia coli* NlpI•Prc complex. However, the NlpI•Prc and LbcA•CtpA complexes are not orthologs. Therefore, growth-rate-dependent control by analogous but dissimilar complexes might be a widely conserved mechanism, and one that could perhaps be targeted for therapeutic intervention.

## INTRODUCTION

The Gram-negative bacterium *Pseudomonas aeruginosa* is widespread in the environment and a significant human pathogen, especially in clinical settings where it causes a range of infections (1). Its intrinsic antibiotic resistance and ability to develop increased resistance to multiple classes of antibiotics are a pressing concern (2, 3). Much of its intrinsic antibiotic resistance can be attributed to the properties of its cell envelope, a multilayered structure that provides a formidable barrier to environmental stresses and antimicrobial agents (4). The cell envelope is composed of three distinct but interlinked macromolecular layers: the inner membrane (IM), the peptidoglycan (PG) cell wall, and the outer membrane (OM) (5). The IM is a symmetric phospholipid bilayer that forms the boundary of the cytoplasm. Surrounding the IM is the PG cell wall, which is composed of linear glycan polymers covalently linked by cross-linked peptide side chains. The OM is asymmetrical, with an inner leaflet of phospholipids and an outer leaflet of lipopolysaccharide. The periplasmic space between the IM and OM is filled with a dense matrix of enzymes and structural proteins that play critical roles in cell envelope synthesis, adaptive responses, and the export and import of various molecules. The processes responsible for the synthesis and maintenance of the cell envelope are critical for viability, and so they must be carefully controlled and coordinated.

Proteolytic enzyme turnover plays a role in the post-translational regulation of biosynthetic pathways, including some of those in the cell envelope (6–8). One widely conserved family of proteases in the cell envelope is the carboxyl-terminal processing proteases (CTPs) (8). *P. aeruginosa* has two CTPs, CtpA and Prc, which belong to distinct CTP subfamilies. Our laboratory began investigating the role of CtpA after discovering that it was essential for type III secretion system function and virulence in a mouse model of acute pneumonia (9). CtpA functions with its partner protein LbcA (lipoprotein-binding partner of CtpA) to degrade at least four predicted PG cross-link endopeptidases, MepM, PA1198, PA1199, and PA4404, and one other cell-wall-associated protein of unknown function, PA1048 (10, 11). LbcA facilitates CtpA-dependent proteolysis by acting as a scaffold for CtpA and its substrates, and by switching CtpA into its active conformation (10, 12). The LbcA•CtpA complex functions similarly to the NlpI•Prc complex of *Escherichia coli*, which also degrades several cell wall lytic enzymes or “autolysins,” including homologs of some of those degraded by LbcA•CtpA in *P. aeruginosa* (6, 13–15). This is remarkable for a couple of reasons. First, the two systems are strikingly different: CtpA and Prc are not orthologs and belong to different CTP subfamilies, the primary sequences of their LbcA- and NlpI-binding partners are not similar, and the macromolecular structures of the LbcA•CtpA and NlpI•Prc complexes are very different (12, 16). Second, even though CtpA accomplishes some of the roles played by Prc in *E. coli*, *P. aeruginosa* also has a Prc ortholog.

Autolysins are potentially dangerous to the bacterial cell, but they are also needed to facilitate cell growth and division. Therefore, there are likely to be mechanisms to control the ability of proteases such as Prc and CtpA to degrade them. It was recognized early on that degradation of the MepS endopeptidase by NlpI•Prc in *E. coli* was reduced when cells were growing rapidly, even though the levels of Prc and NlpI did not change (6). More recently, experiments have suggested that the proteolytic activity of the NlpI•Prc complex is altered in coordination with the rate of phospholipid synthesis to balance membrane and cell wall expansion (17). It was proposed that increased phospholipid export to the OM disrupts its asymmetry, and the removal of mislocalized phospholipids from the outer leaflet of the OM reduces NlpI•Prc activity. The authors hypothesized that a ligand generated by the removal of these phospholipids might engage a binding pocket on NlpI (17).

Studies of CtpA in *P. aeruginosa*, including its biochemical mechanisms and interaction with LbcA, have expanded our understanding of substrate recognition and degradation processes (10, 12, 18, 19). However, there has not been any investigation into regulation within the LbcA•CtpA system *in vivo*. Its major differences from the *E. coli* NlpI•Prc complex mean that it is not obvious that any control mechanisms are likely to be conserved between the two systems/species. For example, not only do the LbcA and NlpI primary sequences lack homology, but LbcA does not have the same potential ligand-binding pocket that is present in NlpI. Therefore, in this study, we set out to begin to investigate whether there is any control in the LbcA•CtpA proteolytic system, and our findings indicate that there is.

## RESULTS

### Analysis of CtpA and LbcA protein levels and gene expression

**Fig 1:**
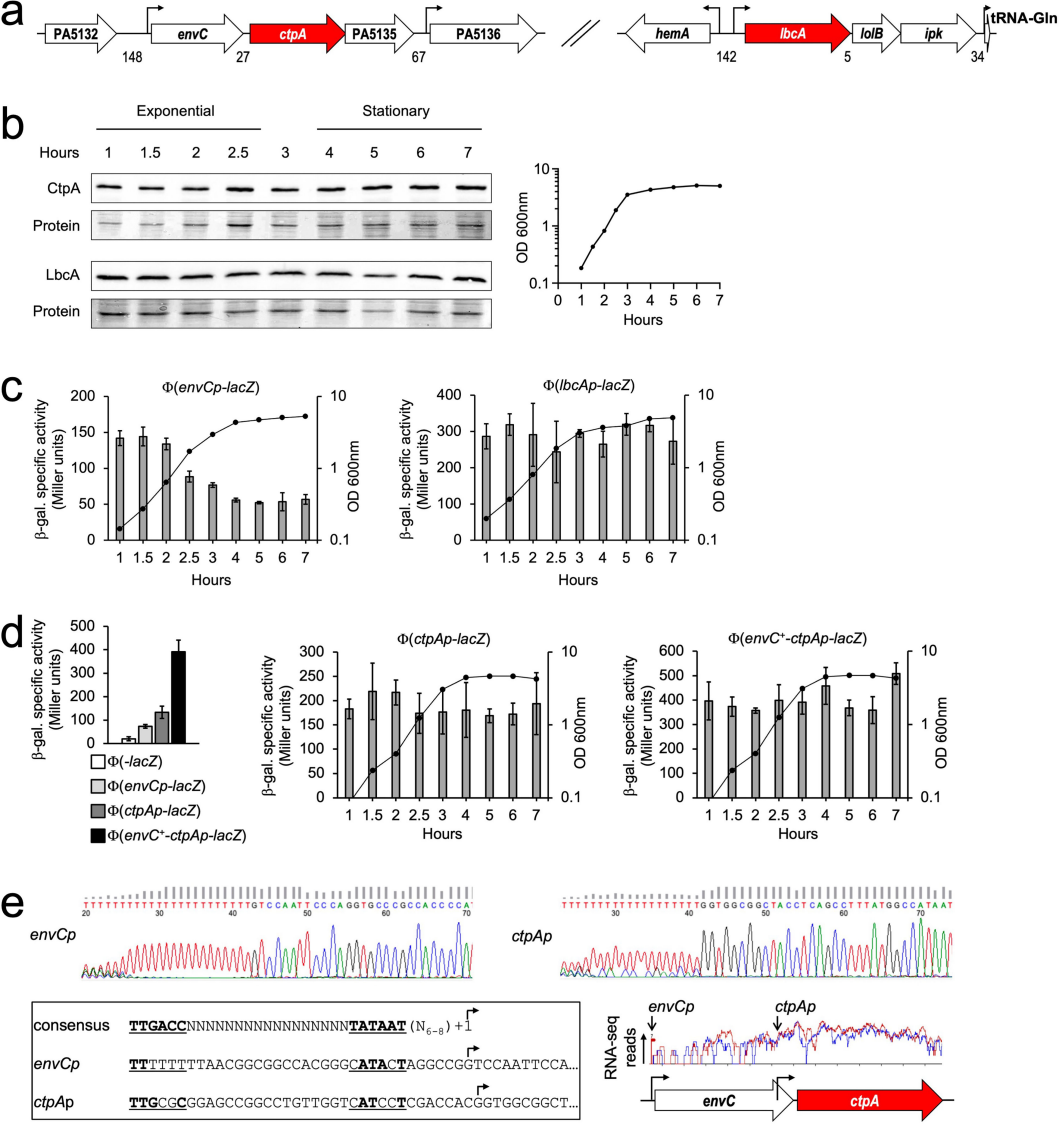
CtpA and LbcA protein levels and gene expression. (**a**) *ctpA* and *lbcA* operons and flanking genes. Right-angled arrows show the locations of TSSs identified by global RNA-sequencing (20). Numbers are intergenic region lengths (bp). (**b**) CtpA and LbcA immunoblot analysis during growth. Protein: Ponceau S-stained region of the immunoblot nitrocellulose membrane (the same region for both immunoblots). The growth curve of the culture is on the right. This panel is a single representative of multiple CtpA and LbcA time course immunoblots, more independent examples of which are in subsequent figures (**c**) Φ(*envCp-lacZ*) and Φ(*lbcAp-lacZ*) operon fusion expression (bars) and growth curves (solid lines). (**d**) Evidence for additional promoter activity immediately upstream of *ctpA*. Φ(*envCp-lacZ*), Φ(*ctpAp-lacZ*), and Φ(*envC^+^-ctpAp-lacZ*) operon fusion expression from cultures in mid-exponential phase (OD_600_ ~1) compared to a strain with no promoter driving *lacZ* expression, Φ(*-lacZ*). Also shown is Φ(*ctpAp-lacZ*) and Φ(*envC^+^-ctpAp-lacZ*) operon fusion expression over time (bars) and growth curves (solid lines). In panels c and d, β-Galactosidase data are averages from three independent cultures (error bars: standard deviation from the mean). Growth curves are single examples of one of the cultures used to generate the β-Galactosidase data. (**e**) Identification of 5′ mRNA ends upstream of *envC* and *ctpA*. The top is raw 5′ RACE DNA sequencing data of cDNA fragments poly A tailed at 5′ ends (reverse complement sequences shown). Bottom left are DNA sequences upstream of each putative TSS, with −10 and −35 motifs in bold underline, and the *P. aeruginosa* consensus sequence (21). Bottom right is mRNA reads for the *envC* operon at 28°C (blue) and 37°C (red) determined by others (20). Locations of 5′ mRNA ends are indicated by downward-facing arrows (*envCp* and *ctpAp*) and by right-angled arrows in the operon diagram.

To determine whether CtpA and/or LbcA abundance is affected by growth rate, we monitored their protein levels and gene expression. Global transcriptome mapping had determined previously that *ctpA* and *lbcA* are in three-gene operons with a single transcription start site (TSS) identified upstream of the first gene in each case (Fig. 1a) (20). Therefore, we constructed strains with single-copy Φ(*envCp-lacZ*) and Φ(*lbcAp-lacZ*) operon fusions to monitor expression of these operons. *envC* is the first gene of the *ctpA* operon (Fig. 1a).

Immunoblot analysis revealed no noticeable change in CtpA and LbcA protein levels at all growth stages (Fig. 1b). Consistent with this, Φ(*lbcAp-lacZ*) expression was also constitutive (Fig. 1c). In contrast*,* Φ(*envCp-lacZ*) expression was threefold lower in the stationary phase compared to the early exponential phase (Fig. 1c). EnvC activates amidases during cell division, a function that fits well with higher *envC* gene expression during rapid growth (22, 23). However, the *envC* promoter is predicted to control the *envC-ctpA*-PA5135 operon, which suggests that *ctpA* expression might also decrease in the stationary phase. This did not fit with the constant CtpA protein levels we observed (Fig. 1b). To explain this, we hypothesized that a promoter within the *envC* operon might drive *ctpA* expression and dampen the influence of the *envC* promoter.

### A promoter within *envC* is responsible for most *ctpA* expression

We constructed two single-copy *lacZ* operon fusion strains to probe for promoter activity upstream of *ctpA*. One had a fragment extending from the *ctpA* start codon to approximately 500 bp upstream of *ctpA*, Φ(*ctpAp-lacZ*). The other had a fragment extending from the *ctpA* start codon to the non-coding region upstream of *envC*, Φ(*envC^+^-ctpAp-lacZ*). Single time point comparison of β-galactosidase activities led to three key observations (Fig. 1d). First, Φ(*ctpAp-lacZ*) expression was higher than a promoterless control, which supported the presence of a promoter in the 500 bp region upstream of *ctpA*. Second, Φ(*ctpAp-lacZ*) expression was higher than Φ(*envCp-lacZ*) expression, suggesting that the *ctpA* promoter was stronger than the *envC* promoter. Third, Φ(*envC^+^-ctpAp-lacZ*) expression was higher than both Φ(*envCp-lacZ*) and Φ(*ctpAp-lacZ*), which is consistent with *lacZ* expression being driven by both the *envC* and *ctpA* promoters. We also analyzed Φ(*ctpAp-lacZ*) and Φ(*envC^+^-ctpAp-lacZ*) expressions at different growth phases, which revealed constitutive expression in both cases (Fig. 1d). All these data suggested that there is a relatively strong constitutive promoter upstream of *ctpA* that dampens the influence of any reduced *envC* promoter activity in the stationary phase.

We used 5′ Rapid Amplification of cDNA Ends (5′ RACE) to test for the predicted 5′ mRNA end upstream of *ctpA*. As a positive control, we also analyzed the region upstream of *envC*, because the *envC* TSS had already been identified in the global transcriptome analysis mentioned above (20). Our 5′ RACE analysis identified the same nucleotide that had been identified as the *envC* TSS (Fig. 1e). As predicted by our operon fusion analysis, 5′ RACE also found a single 5′ mRNA end 239 bp upstream of the *ctpA* start codon, which is within *envC* (Fig. 1e). There was potential −10 and −35 promoter motifs upstream of this putative TSS. In fact, the −35 motif was closer to the consensus sequence than that of the *envC* promoter, which is consistent with *ctpAp* being stronger than *envCp* (Fig. 1e). The location of the putative TSS upstream of *ctpA* also corresponded with the location where an apparent increase in RNA-seq reads occurred within the *envC* operon in the previous global transcriptome analysis (Fig. 1e) (20).

### Analysis of CtpA substrate gene expression and protein levels

**Fig 2:**
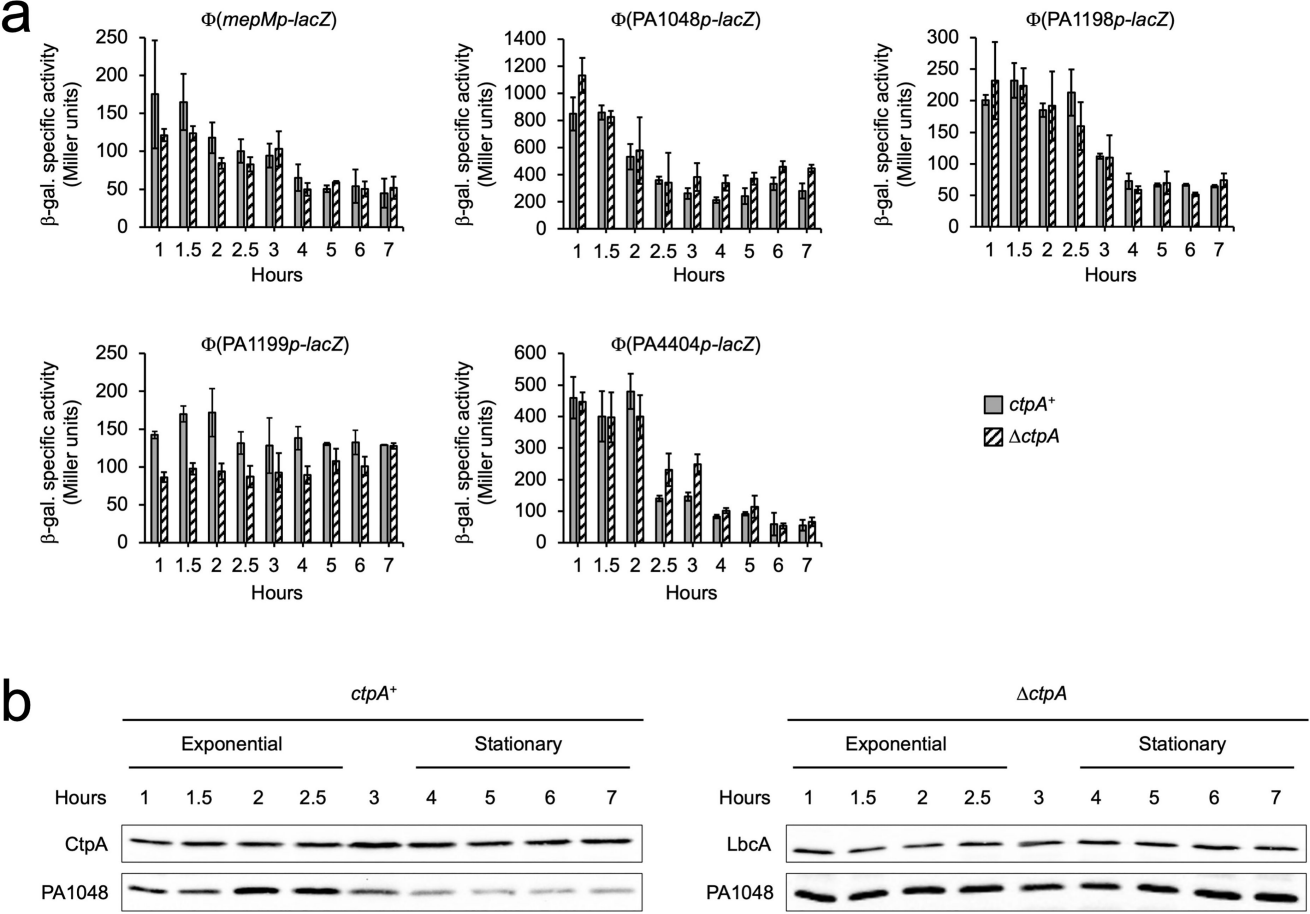
Gene expression and protein level of CtpA substrates. (**a**) *lacZ* operon fusion expression in *ctpA*^+^ and ∆*ctpA* strains. β-Galactosidase data are averages from three independent cultures (error bars: standard deviation from the mean). (**b**) PA1048 Immunoblot analysis during growth. ∆*ctpA* cell extracts were loaded with 1/5th volume of that of the *ctpA*^+^ extracts. Representative experiment showing variation in CtpA substrate level in a *ctpA*^+^ strain, but not a ∆*ctpA* mutant, also observed in multiple similar experiments, including the subsequent figure.

Most CtpA substrates are predicted to be PG cross-link endopeptidases, an activity needed most when cells are growing. Indeed, degradation of the *E. coli* MepS endopeptidase by NlpI•Prc is curtailed during rapid growth (6, 17). Before investigating whether something similar happens for *P. aeruginosa* LbcA•CtpA substrates, we first determined whether substrate gene expression might vary. All CtpA substrate genes are either monocistronic or the first gene of an operon (20). Therefore, we used their non-coding upstream regions to construct *ctpA*^+^ and ∆*ctpA* strains with single-copy *lacZ* operon fusions and analyzed the expression during growth by β-galactosidase assay. The expression of Φ(*mepMp-lacZ*), Φ(PA1048*p-lacZ*), Φ(PA1198*p-lacZ*), and Φ(PA4404*p-lacZ*) displayed the same pattern of trending lower in the stationary phase compared to the early exponential phase (Fig. 2a). This fits with the predicted roles of MepM, PA1198, and PA4404 as PG endopeptidases. In contrast, Φ(PA1199*p-lacZ*) expression was constitutive throughout all growth phases (Fig. 2a). There were only small differences between expression in *ctpA*^+^ and ∆*ctpA* strains.

We also monitored one of the substrate proteins, PA1048. This is the only CtpA substrate for which we have a polyclonal antiserum that can reliably detect the endogenous level in *ctpA*^+^ as well as ∆*ctpA* strains. In a *ctpA*^+^ strain, the PA1048 protein displayed a similar pattern as Φ(PA1048*p-lacZ*) expression, peaking in exponential phase (Fig. 2b). However, in a ∆*ctpA* mutant, the PA1048 level appeared similar at all stages of growth, even though Φ(PA1048*p-lacZ*) expression in a ∆*ctpA* strain was approximately twofold lower in the stationary phase (Fig. 2a and b). Immunoblot analysis might not be sensitive enough to reliably detect the effect of a twofold reduction in gene expression on the PA1048 protein, especially in the ∆*ctpA* strain, where PA1048 was abundant. Regardless, this analysis suggested that any changes in gene expression might play only a minor role in substrate regulation. In contrast, the obvious differences in PA1048 level at different growth phases in a *ctpA*^+^ strain suggested that a change in CtpA-dependent degradation might play the most significant role in controlling substrate levels. We tested this hypothesis next.

### CtpA-dependent proteolysis increases in the stationary phase

**Fig 3:**
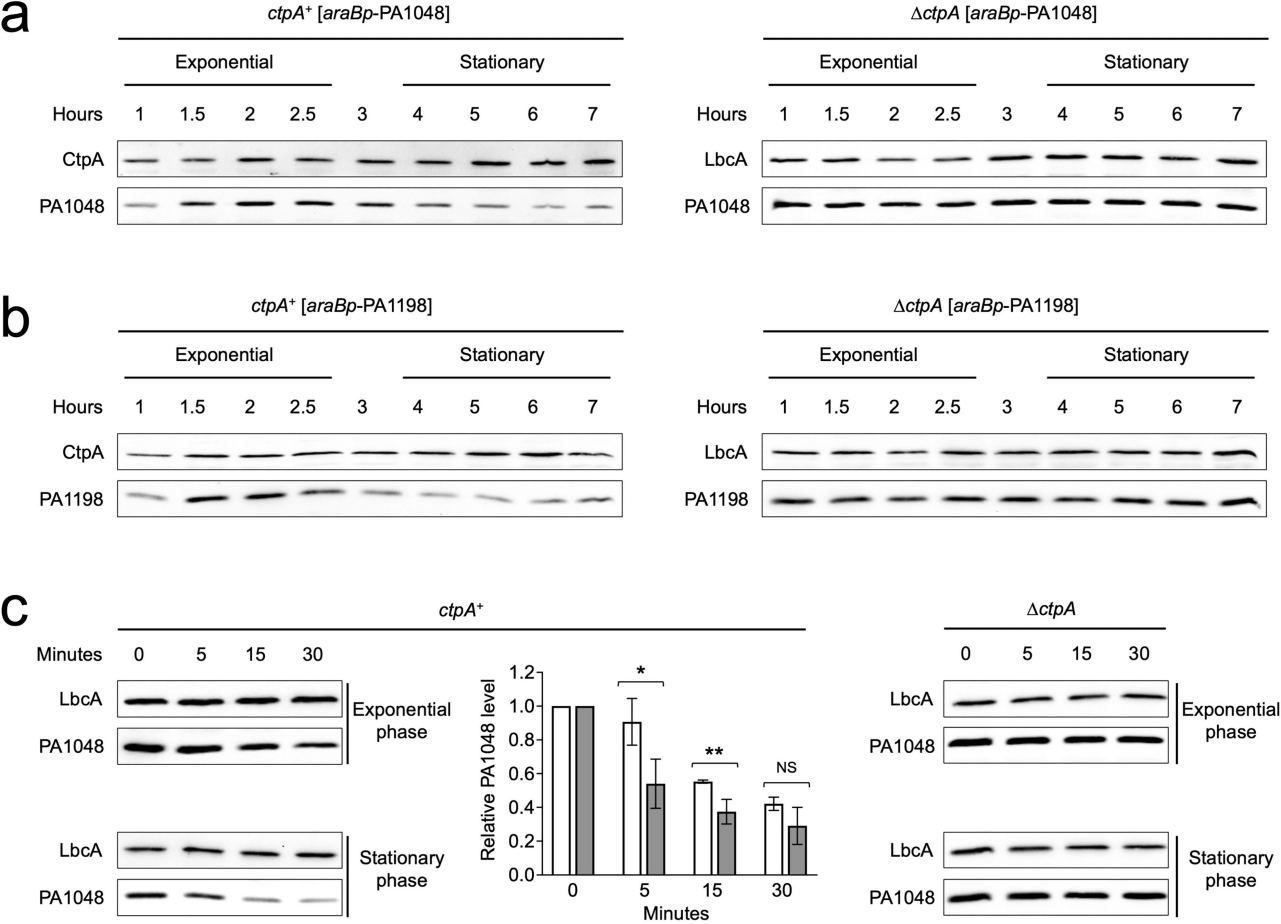
Analysis of CtpA proteolytic activity *in vivo*. (**a**) Immunoblot analysis during growth of ∆PA1048, *ctpA*^+^, and ∆*ctpA* strains containing an *araBp*-PA1048 expression plasmid. PA1048 production was sufficient for detection without arabinose in the culture medium. (**b**) Immunoblot analysis during growth of *ctpA*^+^ and ∆*ctpA* strains containing an *araBp*-PA1198 expression plasmid. The culture medium contained 0.2% arabinose to enable PA1198 detection. Strains were ∆(PA1198-PA1199) to remove endogenous PA1198 and because the antiserum was cross-reactive with PA1199. Strains were also ∆*prc* to eliminate CtpA-independent degradation. Panels a and b are single representative immunoblots of phenomena replicated multiple times. (**c**) PA1048 stability in the exponential and stationary phases in ∆PA0148, *ctpA*^+^, and ∆*ctpA* strains with an *araBp*-PA1048 expression plasmid. Cultures were grown to OD_600_ ~0.3 (exponential phase) or 3 (stationary phase), after which translation was blocked by adding spectinomycin. Samples were taken at the times indicated for immunoblot analysis. Single representative immunoblots for *ctpA*^+^ and ∆*ctpA* strains are shown. For the *ctpA*^+^ strain, the graph shows the means from four independent experiments, including the representative immunoblot. In each experiment, the relative PA1048 level was determined by calculating the PA1048/LbcA ratio after adding spectinomycin, normalized to their ratio at 0 min. Exponential phase, white bars; stationary phase, gray bars. Error bars indicate the standard deviation from the mean. *P  <  0.05; **P  <  0.01; NS, not statistically significant, *P* > 0.05 (unpaired t test). For all panels, ∆*ctpA* cell extracts were loaded with 1/5th volume of that of the *ctpA*^+^ extracts.

We uncoupled the PA1048 gene from its native promoter by expressing it from the *araB* promoter in a multicopy plasmid. PA1048 protein produced from this plasmid peaked in the exponential phase in a *ctpA*^+^ strain but was similar at all growth stages in a ∆*ctpA* mutant (Fig. 3a). This supports the hypothesis that endogenous PA1048 protein-level changes in the preceding experiment were due mostly to changes in CtpA-dependent degradation rather than endogenous gene regulation. To test whether this might be a PA1048-specific phenomenon, we did a similar experiment with another plasmid-encoded substrate, PA1198. Ongoing work in our laboratory has suggested that Prc might contribute to PA1198 degradation, so we analyzed PA1198 levels in ∆*prc ctpA^+^* and *∆prc ∆ctpA* strains. PA1198 protein behaved similarly to PA1048, peaking in the exponential phase only in the *ctpA^+^* strain (Fig. 3b).

Next, we did *in vivo* post-translational substrate stability assays. *ctpA*^+^ and ∆*ctpA* strains containing the plasmid-encoding PA1048 were grown to early exponential or stationary phase, and translation was inhibited with spectinomycin. Substrate stability was then monitored over time by immunoblot analysis. PA1048 was stable at both growth phases in a ∆*ctpA* mutant, whereas it was unstable in a *ctpA*^+^ strain (Fig. 3c). PA1048 appeared to be slightly more stable in the exponential phase compared to the stationary phase, and quantification of multiple replicate experiments supported that conclusion (Fig. 3c). Therefore, these data support the hypothesis that CtpA activity is lower when cells are growing rapidly and higher in the stationary phase.

### Overexpression of the gene-encoding DedA family protein PA5244 (YohD) reduces CtpA activity *in vivo*

**Fig 4:**
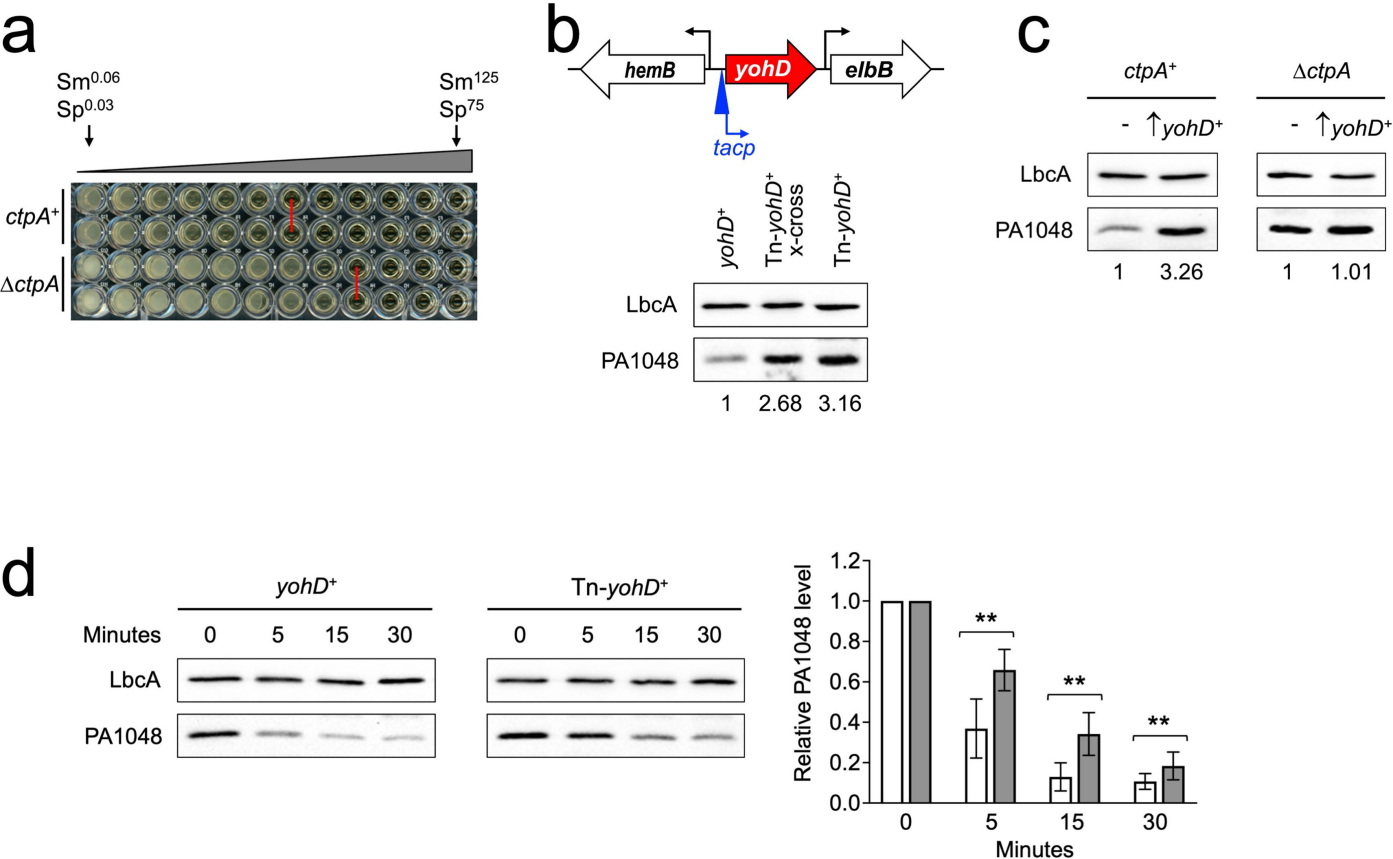
Overexpression of *yohD* (PA5244) reduces CtpA activity. (**a**) Sm/Sp resistance profiles of *ctpA*^+^ and ∆*ctpA* Φ(PA3923p-*lacZ*) operon fusions strains. Saturated cultures were diluted to approximately 5 × 10^5^ colony-forming units per mL in a volume of 200 µL in the wells of a 96-well microtiter plate. Wells contained twofold serial dilutions of Sm/Sp from the highest concentration of Sm 125 mg/mL and Sp 75 µg/ml in the wells on the right-hand side. Each strain was tested in duplicate. Wells with the lowest antibiotic concentrations that inhibited all visible growth after 18 hours of incubation at 37°C are indicated by red bars. (**b**) Top: genomic arrangement of *yohD* with right-angled arrows showing transcription start sites identified by global RNA-sequencing (20) and the approximate location of the transposon insertion with its outward-facing *tac* promoter in blue. Bottom: immunoblot analysis of steady-state protein levels in the stationary phase. *yohD*^+^: parental strain (*yohD*^+^). Tn-*yohD*^+^: mutant with transposon insertion immediately upstream of *yohD*. Tn-*yohD*^+^ x-cross: transposon insertion backcross strain. Numbers at the bottom show the PA1048/LbcA ratio relative to the ratio in the parental strain. (**c**) *ctpA*^+^ and ∆*ctpA* strains with an *araBp-yohD* expression plasmid (↑*yohD*^+^) or the empty vector (-) were grown to the stationary phase and analyzed by immunoblot. ∆*ctpA* cell extracts were loaded with 1/5th volume of that of the *ctpA*^+^ extracts. Numbers at the bottom show the PA1048/LbcA ratio in each ↑*yohD*^+^ strain relative to the corresponding empty vector strain. (**d**) PA1048 stability analysis. The parental strain (*yohD*^+^) or the mutant with a transposon insertion immediately upstream of *yohD* (Tn-*yohD*^+^) was grown to the stationary phase, after which translation was blocked by adding spectinomycin. Samples were taken at the times indicated for immunoblot analysis. Single representative immunoblots for each strain are shown. The graph shows the means from six (*yohD*^+^) or seven (Tn-*yohD*^+^) independent experiments, including the representative immunoblots. Relative PA1048 level was determined by calculating the PA1048/LbcA or CtpA ratio after adding spectinomycin, normalized to their ratio at 0 min in each experiment. *yohD*^+^, white bars; Tn-*yohD*^+^, gray bars. Error bars indicate the standard deviation from the mean. **P  <  0.01 (unpaired t test).

To begin to explore how LbcA•CtpA complex activity is controlled, we attempted to find transposon insertion mutants with reduced CtpA activity. An unrelated study in our laboratory has discovered that the expression of the PA3923 gene, encoding a predicted OM protein of unknown function, is upregulated in a *ctpA* null mutant. A ∆*ctpA* mutant with a single-copy Φ(PA3923*p-aadA*) operon fusion was more streptomycin/spectinomycin (Sm/Sp) resistant than a *ctpA*^+^ strain (*aadA* encodes Sm/Sp resistance) (Fig. 4a). Therefore, this fusion indirectly reports reduced CtpA activity. We isolated transposon mutants with increased Sm/Sp resistance, analyzed them by anti-PA1048 substrate immunoblot to find those likely to have reduced CtpA activity, and by anti-CtpA/LbcA immunoblot to eliminate any *ctpA* or *lbcA* mutants. We used a transposon with an outward-facing *tac* promoter to identify null or overexpression mutations. Using a high Sm/Sp concentration to screen for increased Sm/Sp resistance identified probable *ctpA* null mutants (no CtpA protein detected by immunoblot), which validated the approach, but was not informative. However, reducing the stringency led to the repeated identification of a mutant with a transposon inserted immediately upstream of PA5244 (*yohD*), encoding a member of the DedA family implicated as lipid and phospholipid flippases (Fig. 4b) (24, 25). The phenotype was reproduced when the transposon insertion was backcrossed into the parental strain, confirming that it was not caused by a spurious mutation (Fig. 4b).

Global transcriptome analysis had indicated that endogenous *yohD* expression is low in the laboratory (20). However, the transposon had inserted 33 bp upstream of the *yohD* start codon in an orientation suggesting that its outward-facing *tac* promoter might have caused *yohD* overexpression. To test whether *yohD* overexpression reduces CtpA activity, we determined the effect of plasmid-encoded *yohD* expression on the level of PA1048 in *ctpA*^+^ and ∆*ctpA* strains. *yohD* overexpression increased the level of PA1048 in a *ctpA*^+^ strain but not in a ∆*ctpA* strain, supporting the hypothesis that it might reduce CtpA activity (Fig. 4c). Post-translational substrate stability assays supported this conclusion by revealing that the transposon insertion upstream of *yohD* caused a modest reduction in the rate of PA1048 degradation (Fig. 4d).

### Overproduction of AsmA family phospholipid bridge(s) reduces CtpA activity *in vivo*

**Fig 5:**
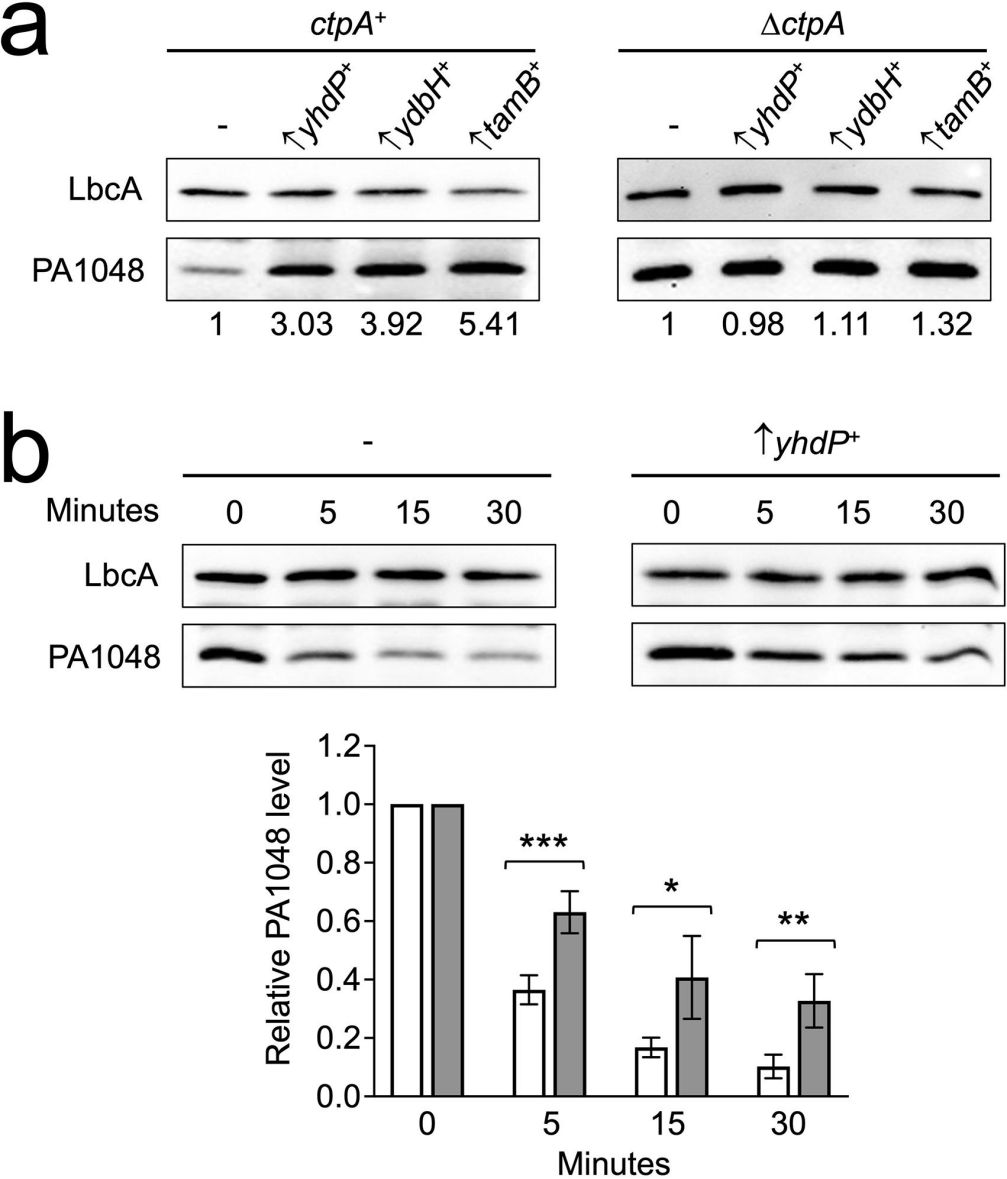
Overexpression of genes encoding AsmA-family members reduces CtpA activity. (**a**) *ctpA*^+^ and ∆*ctpA* strains with *araBp* expression plasmids encoding the indicated genes (↑) or the empty vector (-) were grown to the stationary phase and analyzed by immunoblot. ∆*ctpA* cell extracts were loaded with 1/5th volume of that of the *ctpA*^+^ extracts. Numbers at the bottom show the PA1048/LbcA ratio relative to the corresponding empty vector strain. (**b**) PA1048 stability analysis. *ctpA*^+^ strains with an *araBp-yhdP* expression plasmid (↑*yhdP*^+^) or the empty vector (-) were grown to the stationary phase, after which translation was blocked by adding spectinomycin. Samples were taken at the times indicated for immunoblot analysis. Single representative immunoblots for each strain are shown. The graph shows the means from four independent experiments for each strain, including the representative immunoblots. Relative PA1048 level was determined by calculating the PA1048/LbcA or CtpA ratio after adding spectinomycin, normalized to their ratio at 0 min in each experiment. Empty vector, white bars; *araBp-yhdP*, gray bars. Error bars indicate the standard deviation from the means. *P  <  0.05; **P  <  0.01; ***P  <  0.001 (unpaired t test).

DedA family members implicated as IM phospholipid flippases are hypothesized to interface with AsmA-type transporters to form trans-envelope bridges for phospholipid transport (24–28). *yohD* overexpression might reduce CtpA activity because it increases phospholipid flipping across the IM for transport to the OM. Indeed, increased phospholipid transport to the OM has been implicated in downregulating the *E. coli* NlpI•Prc complex (17). This led us to hypothesize that overproduction of some AsmA proteins might also reduce CtpA activity. To test this, we focused on three conserved AsmA-like proteins that have been suggested to transport phospholipids to the OM in both *E. coli* and *P. aeruginosa*: PA2542 (*tamB*), PA4476 (*yhdP*), and PA5307 (*ydbH)* (27–29). Consistent with our hypothesis, plasmid-encoded expression of *tamB*, *yhdP,* or *ydbH* increased the steady-state level of the PA1048 substrate in *ctpA*^+^ strains but not in ∆*ctpA* strains (Fig. 5a). Stability assays with one representative further supported this conclusion by revealing that *yhdP* overexpression increased the post-translational stability of PA1048 (Fig. 5b).

### Overproduction of two proteins implicated in removing and destroying mislocalized phospholipids from the OM reduces CtpA activity *in vivo*

**Fig 6:**
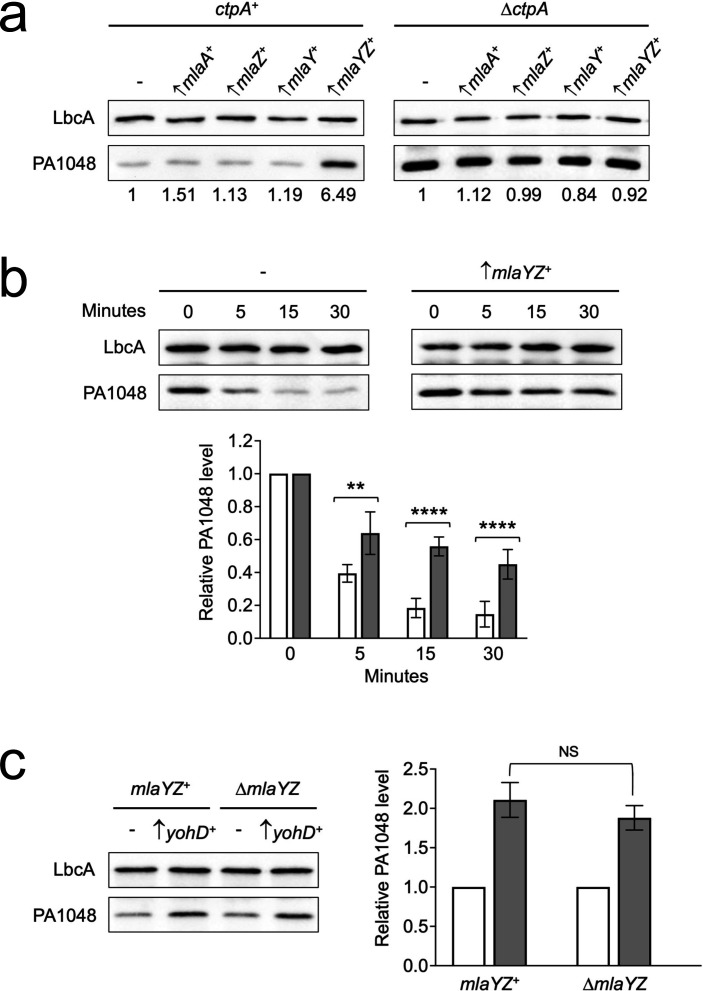
Overexpression of *mlaYZ* reduces CtpA activity. (**a**) *ctpA*^+^ and ∆*ctpA* strains with *araBp* expression plasmids containing the indicated genes (↑) or the empty vector (-) were grown to the early stationary phase and analyzed by immunoblot. ∆*ctpA* cell extracts were loaded with 1/5th volume of that of the *ctpA*^+^ extracts. Numbers at the bottom show the PA1048/LbcA ratio relative to the corresponding empty vector strain. (**b**) PA1048 stability over time. *ctpA*^+^ strains with an *araBp-mlaYZ* expression plasmid (↑*mlaYZ*^+^) or the empty vector (-) were grown to the stationary phase after which translation was blocked by adding spectinomycin. Samples were taken at the times indicated for immunoblot analysis. Single representative immunoblots for each strain are shown. The graph shows the means from four independent experiments for each strain, including the representative immunoblots. Relative PA1048 level was determined by calculating the PA1048/LbcA ratio after adding spectinomycin, normalized to their ratio at 0 min in each experiment. Empty vector, white bars; *araBp-mlaYZ*, gray bars. Error bars indicate the standard deviation from the mean. **P  <  0.01; ****P  <  0.0001 (unpaired t test). (**c**) *mlaYZ*^+^ and ∆*mlaYZ* strains with an *araBp-yohD* expression plasmid (↑*yohD*^+^) or the empty vector (-) were grown to the stationary phase and analyzed by immunoblot. A single representative immunoblot is shown. The graph shows the means from four independent experiments for each strain, including the representative immunoblot. For each strain, the relative PA1048 level was determined by calculating the PA1048/LbcA ratio with *yohD* overexpression normalized to the ratio with the Empty vector. Empty vector, white bars; *araBp-yohD*, gray bars. Error bars indicate the standard deviation from the mean. NS, not statistically significant, *P* > 0.05 (unpaired t test).

During rapid growth, increased phospholipid transport to the *E. coli* OM causes mislocalization into the outer leaflet, with the subsequent removal of these phospholipids proposed to signal the NlpI•Prc complex to reduce its activity (17). Our finding that increased production of proteins implicated in transporting phospholipids correlated with reduced CtpA activity suggested that a similar phenomenon might downregulate LbcA•CtpA activity (Fig. 4 and 5). Therefore, we extended our analysis by testing whether some proteins involved in removing mislocalized phospholipids from the *P. aeruginosa* OM affected CtpA activity. Two MlaA-family OM proteins have been implicated in this process in *P. aeruginosa*: PA2800/MlaA is part of a canonical Mla pathway involved in retrograde phospholipid transport, whereas PA3239/MlaZ was proposed to transfer mislocalized phospholipids to the lipase PA3238/MlaY for destruction (30). Overexpression of *mlaA, mlaZ,* or *mlaY* in isolation had no obvious effect on the steady-state level of CtpA substrate PA1048 (Fig. 6a). However, *mlaYZ* co-overexpression led to a several-fold increase in the level of PA1048 in a *ctpA*^+^ strain but not in a ∆*ctpA* strain. Protein stability assays further supported the conclusion that *mlaYZ* overexpression reduced CtpA activity by revealing that *mlaYZ* overexpression caused a robust increase in PA1048 post-translational stability (Fig. 6b).

Our data suggested that CtpA activity might be reduced when increased transport of phospholipids causes their mislocalization into the outer leaflet of the OM and subsequent removal and destruction by MlaYZ. Therefore, in our final experiment, we tested whether overexpression of *yohD*, encoding the putative DedA family lipid/phospholipid flippase, could still reduce CtpA activity in a ∆*mlaYZ* strain. Initial experiments suggested that the ∆*mlaYZ* mutation had either no effect or a minimal effect. To increase rigor, we quantified multiple independent replicates, and the data showed that a ∆*mlaYZ* mutation has no statistically significant effect on the ability of *yohD* overexpression to reduce CtpA activity (Fig. 3c; see Discussion).

## DISCUSSION

We have investigated the control of the LbcA•CtpA proteolytic complex and its substrates. In the stationary phase, four of the five substrate-encoding genes had modestly reduced expression, and the substrate proteins had increased CtpA-dependent degradation (Fig. 2). We did not pursue the mechanism by which the four substrate genes might be downregulated in the stationary phase. We used the Miller method to measure β-galactosidase activities, which uses OD of cell suspensions for normalization (31, 32). This method is most typically used to measure gene expression in the exponential phase, due to concerns about reduced metabolic activity, protein levels, and gene expression heterogeneity in the stationary phase. These caveats should be considered when interpreting our data. However, the Miller method has been used before to monitor gene expression in different growth phases (e.g., 33–35). Also, our substrate promoter *lacZ* operon fusion strains were identical except for the promoter driving *lacZ* expression at the neutral *attB* site. Therefore, the only obvious explanation for the observation that most had reduced β-galactosidase activity in stationary phase, but Φ(PA1199-*lacZ*) did not, is different expression patterns of the substrate gene promoters (Fig. 2). The genes encoding LbcA and CtpA were constitutively expressed, and their protein levels were also constant in different growth phases (Fig. 1). However, CtpA proteolytic activity was lower when cells were growing rapidly, and our data suggest that its activity might be connected to phospholipid trafficking in the cell envelope. It is intriguing that a similar phenomenon has been reported to affect the *E. coli* NlpI•Prc complex, even though there are major differences between the NlpI•Prc and LbcA•CtpA proteolytic systems (6, 12, 16, 17).

*lbcA* is the first gene of its operon, and its promoter activity was unaffected by growth phase. Constitutive expression of *ctpA* had a more complicated explanation because the *envC-ctpA*-PA5135 operon has a complex arrangement. *envC* promoter activity was higher in the exponential phase, consistent with EnvC being needed during rapid growth to facilitate cell division as an amidase activator (Fig. 1c). *ctpA* appears to be insulated from lower *envC* promoter activity in the stationary phase by a strong constitutive promoter located within *envC* (Fig. 1d and e). The *yibQ/sddA E. coli* ortholog of PA5135, which is the final gene of the *envC* operon, encodes a PG deacetylase involved in cell division (36) (in *E. coli, envC* and *sddA* are adjacent, whereas *ctpA* is between them in *P. aeruginosa*). We did not investigate PA5135 expression, but it might be beneficial for it to be reduced in the stationary phase if it plays a role in cell division. The constitutive *ctpA* promoter could prevent this, but perhaps the *envC-ctpA*-PA5135 operon has additional features to facilitate reduced PA5135 expression in the stationary phase. Global transcriptome analysis data did suggest that PA5135 expression might be lower than *ctpA* (20). Alternatively, data suggest that *E. coli* SddA is a Prc substrate, meaning that proteolysis could provide a transcription-independent mechanism to reduce its level in the stationary phase (36). However, we do not have any data to indicate that PA5135 is a protease substrate in *P. aeruginosa*.

Degradation of CtpA substrates occurred at a higher rate in the stationary phase despite no increase in the levels of CtpA or LbcA (Fig. 1 and 3). This suggested that CtpA activity is subject to post-translational regulation. To test this in a non-biased manner, we screened for transposon mutants with reduced CtpA activity, leading to the finding that PA5244 (*yohD*) overexpression reduced the rate of CtpA-dependent degradation (Fig. 4). YohD is a predicted member of the DedA family of proposed IM phospholipid flippases (24, 25). The flippase activity of some DedA proteins is thought to be an early step in the transfer of phospholipids to the OM, in conjunction with AsmA family members that form trans-envelope phospholipid bridges (24–28). Increased phospholipid synthesis and transport to the OM in *E. coli* has been proposed to generate a signal that downregulates activity of the NlpI•Prc complex (17). Therefore, instead of extending our transposon mutant screen to saturation, something we hope to do in the future, we followed our discovery of the *yohD* overexpression effect by exploring the possibility of a parallel between *E. coli* NlpI•Prc and *P. aeruginosa* LbcA•CtpA regulation.

In further support of similar mechanisms controlling NlpI•Prc and LbcA•CtpA, we found that CtpA activity was reduced by overproduction of three AsmA-family proteins implicated in phospholipid transport (Fig. 5). In *E. coli,* phospholipid synthesis and transport are high during the exponential phase and low in the stationary phase (27, 37, 38). If the same is true in *P. aeruginosa,* the rate of phospholipid transport to the OM could provide a growth rate-dependent signal to control LbcA•CtpA complex activity. Rapid phospholipid transport to the *E. coli* OM in the exponential phase causes mislocalization into the outer leaflet, and experiments suggested that this was involved in signaling the NlpI•Prc complex to reduce its activity (17). It was proposed that PldA phospholipase-mediated destruction of these mislocalized phospholipids might generate a ligand that binds to NlpI and reduces Prc activity. Our experiments were consistent with a similar scenario for LbcA•CtpA because overproduction of the MlaYZ proteins reduced the rate of CtpA-dependent degradation (Fig. 6). MlaZ is an OM MlaA-family protein proposed to extract phospholipids from the outer leaflet of the OM and pass them to the MlaY phospholipase for destruction (30). However, we also found that a ∆*mlaYZ* mutation did not prevent overexpression of *yohD*, encoding a putative IM lipid/phospholipid flippase, from reducing CtpA activity (Fig. 6c). Even if YohD can increase phospholipid flow to the OM and mislocalization into the outer leaflet, there might be other proteins besides MlaYZ that can remove and/or degrade them to generate a signal/condition to influence CtpA activity, such as PlpD or other uncharacterized proteins (39). Alternatively, there might be more to the mechanism by which YohD and the other proteins we found affect CtpA activity that we do not understand. Related to this, the functions of YohD, YhdP, YdbH, and TamB are not well characterized in *P. aeruginosa*. Also, *E. coli* YhdP, YdbH, and TamB are predicted to facilitate bidirectional diffusive flow of phospholipids, not necessarily unidirectional to the OM (27). Therefore, their overproduction might disrupt phospholipid transport rather than accelerate it. This still might disrupt OM integrity/asymmetry, but the uncertainty about their effects in *P. aeruginosa* means that various scenarios for how CtpA activity could be affected are possible.

Rapid growth, or overexpression of genes characterized in this study, reduced the rate of CtpA-dependent proteolysis *in vivo* (Fig. 3c, 4d, 5b and 6b). However, this reduction in CtpA activity was less than the complete loss of activity in a ∆*ctpA* null strain. In immunoblots, ∆*ctpA* extracts were loaded with 1/5th volume of the *ctpA*^+^ extracts, because substrate accumulation was much higher in the ∆*ctpA* strain. Also, a CtpA substrate was still degraded during rapid growth or upon gene overexpression (Fig. 3c, 4d, 5b and 6b). Therefore, we would not expect the overexpression of *yohD*, *ydhP*, *ydbH*, *tamB*, or *mlaYZ* in a *ctpA*^+^ strain to phenocopy ∆*ctpA* mutant phenotypes, and we have no evidence that they do so. Similarly, when a link between phospholipid transport/OM mislocalization and the *E. coli* NlpI•Prc complex was discovered, protease-defective cell extracts (∆*nlpI*) were underloaded at 1/5th volume, and also no effects on Prc-dependent phenotypes were reported (17).

Growth-rate-dependent regulation of *E. coli* Prc activity was proposed to work through its NlpI lipoprotein binding partner (6, 14, 17). For example, Prc can cleave some proteins without the involvement of NlpI, but this was unaffected by growth rate (17). Control of CtpA activity might also act via its LbcA binding partner, but we could not test this because the degradation of all five known CtpA substrates requires LbcA (10, 11). A depression was noted on one side of each monomer in the NlpI dimer structure, which was suggested to be a potential ligand-binding pocket lined by hydrophobic acids (40). This was implicated as the possible binding site for a ligand generated from phospholipase-mediated degradation of phospholipids (17). Something similar might happen with LbcA in *P. aeruginosa*. However, LbcA and NlpI are not homologous. Despite this, like NlpI, LbcA also contains a domain composed of multiple tetratricopeptide repeat (TPR) motifs, which are known to function in ligand binding (41, 42). Most known TPR domain ligands are peptides involved in forming protein-protein interactions, but there is also precedent for a fatty acid ligand (43, 44). Therefore, the potential for LbcA binding to a phospholipid-derived ligand remains a possibility that could be further pursued in the future. More recently, the predicted inactive M16 metallopeptidase family member BipP was also identified as a negative regulator of *E. coli* Prc, by binding to NlpI (45). The possibilities that NlpI•Prc is controlled by a phospholipid-derived ligand and by BipP are not mutually exclusive. They could act as independent signal inputs, or both might be part of the same control pathway. *P. aeruginosa* does not have a close sequence homolog of BipP, although it does have members of the same family.

As noted earlier, there are striking differences between the *E. coli* NlpI•Prc and *P. aeruginosa* LbcA•CtpA proteolytic complexes. Therefore, it is remarkable that these two different complexes play similar roles, especially when considering the additional fact that *P. aeruginosa* also has a Prc ortholog. This study has revealed another similarity between these dissimilar complexes: the activity of each varies according to growth rate, and this might have a similar underlying mechanism. Obviously, many questions remain regarding the mechanistic details of exactly how this control is achieved. Regardless, it seems likely that growth-rate-dependent autolysin control by CTP-containing proteolytic complexes is widely conserved in bacteria, even though it is achieved by non-orthologous proteins in some cases.

## MATERIALS AND METHODS

### Bacterial strains and growth conditions

Bacterial strains and plasmids are listed in Table 1. Routine growth of bacterial cultures was in Luria-Bertani (LB) broth, composed of 1% (wt/vol) tryptone, 0.5% (wt/vol) yeast extract, and 1% (wt/vol) NaCl or on LB agar. For some procedures involving *P. aeruginosa*, Vogel-Bonner minimal (VBM) agar was used as a selective growth medium. *E. coli* K-12 strain SM10 served as the donor to conjugate plasmids into *P. aeruginosa* (46). Routine antibiotic concentrations used were as follows: ampicillin (200 µg/mL fo*r E. coli*), tetracycline (15 µg/mL for *E. coli*, 75 µg/mL for *P. aeruginosa*), gentamicin (15 µg/mL for *E. coli*, 75 µg/mL for *P. aeruginosa*), streptomycin (50 µg/mL for *E. coli*, 250 µg/mL for *P. aeruginosa*), and spectinomycin (50 µg/mL for *E. coli*, 500 µg/mL for *P. aeruginosa*).

**TABLE 1:** Strains and plasmids

Name	Genotype/features	Reference/source
*P. aeruginosa* strains		
PAK	Parental reference strain^*a*^	(47)
AJDP730	∆*ctpA*	(9)
AJDP1343	*attB*::[Φ(PA1199*p-lacZ*)]	This study
AJDP1344	*attB*::[Φ(PA1199*p-lacZ*)] ∆*ctpA*	This study
AJDP1345	*attB*::[Φ(*mepMp-lacZ*)]	This study
AJDP1346	*attB*::[Φ(*mepMp-lacZ*)] ∆*ctpA*	This study
AJDP1376	*attB*::[Φ(PA4404*p-lacZ*)]	This study
AJDP1377	*attB*::[Φ(PA4404*p-lacZ*)] ∆*ctpA*	This study
AJDP1385	∆(PA1198-PA1199) ∆*prc*	(19)
AJDP1386	∆(PA1198-PA1199) ∆*prc* ∆*ctpA*	(19)
AJDP1389	∆(PA1198-PA1199)	(19)
AJDP1390	∆(PA1198-PA1199) ∆*ctpA*	(19)
AJDP1395	*attB*::[Φ(PA1198*p-lacZ*)]	This study
AJDP1396	*attB*::[Φ PA1198*p-lacZ*)] ∆*ctpA*	This study
AJDP1546	∆PA1048	This study
AJDP1549	∆PA1048 ∆*ctpA*	This study
AJDP1593	*attB*::[Φ(PA1048*p-lacZ*)]	This study
AJDP1688	*attB*::[Φ(*envCp-lacZ*)]	This study
AJDP1699	*attB*::[Φ(PA1048*p-lacZ*)] ∆*ctpA*	This study
AJDP1716	*attB*::[Φ(*ctpAp-lacZ*)]	This study
AJDP1717	*attB*::[Φ(*lbcAp-lacZ*)]	This study
AJDP1725	*attB*::[Φ(*envC^+^-ctpAp-lacZ*)]	This study
AJDP1770	∆*mlaYZ*	This study
AJDP1788	*attB*::[Φ(PA3923*p-aadA1*)] *Tn-yohD^+b^*	This study
AJDP1789	*attB*::[Φ(PA3923*p-aadA1*)] *Tn-yohD^+^* (backcross strain)^*c*^	This study
AJDP1798	*attB*::[Φ(PA3923*p-aadA1*)]	This study
AJDP1799	*attB*::[Φ(PA3923*p-aadA1*)] ∆*ctpA*	This study
Plasmids		
pBT20	*mariner* Tn delivery plasmid, R6K *ori*	(48)
pCP20	FLP recombinase plasmid	(49)
pEX18Ap	Amp^R^, pMB1 *ori, oriT, sacB^+^*	(50)
pHERD20T	Amp^R^, pMB1 *ori, araBp* expression vector	(51)
pHERD26T	Tet^R^, pMB1 *ori, araBp* expression vector	(51)
mini-CTX-lacZ	Tet^R^, pMB1 *ori, oriT, int^+^, attP^+^, lacZ*^+^	(52)
pVLT35	Sm^R^ Sp^R^, RSF1010 *ori, tacp* expression vector	(53)
pAJD2916	*araBp*-PA1198 in pHERD20T	This study
pAJD2925	Φ(PA1199*p-lacZ*) in mini-CTX-lacZ	This study
pAJD2926	Φ(*mepMp-lacZ*) in mini-CTX-lacZ	This study
pAJD2938	Φ(PA4404*p-lacZ*) in mini-CTX-lacZ	This study
pAJD2960	Φ(PA1198*p-lacZ*) in mini-CTX-lacZ	This study
pAJD2963	Φ(PA3923*p-lacZ*) in mini-CTX-lacZ	This study
pAJD3065	*araBp*-PA1048 in pHERD26T	This study
pAJD3070	Φ(PA1048*p-lacZ*) in mini-CTX-lacZ	This study
pAJD3175	Φ(*envCp-lacZ*) in mini-CTX-lacZ	This study
pAJD3265	Φ(*ctpAp-lacZ*) in mini-CTX-lacZ	This study
pAJD3266	Φ(*lbcAp-lacZ*) in mini-CTX-lacZ	This study
pAJD3267	Φ(*envC*^+^*ctpAp-lacZ*) in mini-CTX-lacZ	This study
pAJD3286	*araBp-mlaA* in pHERD26T	This study
pAJD3287	*araBp-mlaY* in pHERD26T	This study
pAJD3288	*araBp-mlaYZ* in pHERD26T	This study
pAJD3298	*araBp-mlaZ* in pHERD26T	This study
pAJD3303	*araBp-yohD* in pHERD26T	This study
pAJD3305	*araBp-yhdP* in pHERD26T	This study
pAJD3313	*araBp-ydbH* in pHERD26T	This study
pAJD3314	*araBp-tamB* in pHERD26T	This study
pAJD3315	Φ(PA3923*p-aadA1*) in mini-CTX-∆lacZ	This study

^*a*^ Alltrains are derivatives of strain PAK, but the most frequently used PA gene reference numbers from strain PAO1 are shown.

^*b*^ *yohD* = PA5244. The transposon is inserted between nucleotides T-6028327 and A-6028328 of the PAK genome (54).

^*c*^ The transposon insertion of strain AJDP1788 was backcrossed into AJDP1798.

### Strain and plasmid constructions

To construct in-frame deletion mutants, fragments approximately 0.55 kb in size flanking the regions upstream and downstream of the deletion site were amplified by PCR. The PCR products were cloned into pEX18Ap using restriction sites incorporated by the PCR primers. The plasmid was integrated into the *P. aeruginosa* chromosome after conjugation from *E. coli*. Sucrose-resistant, carbenicillin-sensitive segregants were isolated on LB agar containing 10% sucrose without NaCl. The presence of the deletion was verified by PCR analysis of genomic DNA.

Expression plasmids were constructed by amplifying genes from *P. aeruginosa* chromosomal DNA using one primer that annealed ~30 bp upstream of the start codon and a second primer that annealed immediately downstream of the stop codon. The amplified fragments were cloned into pHERD20T or pHERD26T using restriction sites incorporated by the PCR primers.

Single-copy *lacZ* operon fusion strains were made by amplifying the entire noncoding region upstream of each gene, sometimes with additional upstream DNA as indicated in the text, and cloning them into the mini-CTX-*lacZ* vector. The plasmids were integrated into the *attB* site of the *P. aeruginosa* chromosome, backbone vector DNA was removed by pFLP2-mediated excision, and the integrations were confirmed by colony PCR analysis as described (52).

To construct the Φ(*PA3923p-aadA1*) operon fusion strain, first, a Φ(*PA3923p-lacZ*) operon fusion plasmid was constructed by amplifying the PA3923 upstream non-coding region from *P. aeruginosa* PAK genomic DNA. The PCR product was cloned into the mini-CTX-lacZ vector. Next, the *aadA1* streptomycin/spectinomycin resistance gene without its promoter was amplified from plasmid pVLT35 and cloned into the Φ(*PA3923p-lacZ*) operon fusion plasmid between the BamHI/SacI sites, replacing the *lacZ* gene. This plasmid was integrated into the *attB* site of the *P. aeruginosa* chromosome. In this case, the backbone vector DNA was not removed by pFLP2-mediated excision. Integration was confirmed by colony PCR analysis (52).

### β-Galactosidase assays

Saturated cultures were diluted into 25 mL of LB broth in a 125 mL flask so that the optical density (OD)_600_ was approximately 0.05. The cultures were grown in a shaker (225 rpm) at 37°C for 7 h. Aliquots were removed at different times, and the cells were collected by centrifugation. β-Galactosidase enzyme activity was determined at room temperature (approximately 22°C) in permeabilized cells as described previously (31). Activities are expressed in arbitrary Miller units (32). Individual cultures were assayed in duplicate, and reported values are averaged from three independent cultures.

### Determination of *ctpA* and *envC* 5′ mRNA ends

*P. aeruginosa* PAK was grown in LB broth until the OD_600_ was ~1.0. RNA was extracted using the High-Pure RNA isolation kit as described by the manufacturer (Roche). Traces of contaminating DNA were eliminated by a 30 min treatment with Turbo DNA-free DNase as described by the manufacturer (Thermofisher). The absence of contaminating DNA was confirmed by failure to amplify *ctpA* and *envC* by PCR. 5′ mRNA ends upstream of *ctpA* and *envC* were determined using the 5′ RACE System for Rapid Amplification of cDNA Ends, version 2.0, according to the manufacturer’s instructions (Thermofisher).

### Determination of steady-state protein levels and CtpA substrate stability *in vivo*

For time course steady-state protein-level experiments, saturated cultures were diluted into 25 mL of LB broth in a 125 mL flask to an initial OD_600_ of 0.05 and incubated at 37˚C with shaking at 225 rpm. Samples were collected at regular intervals for 7 h. For single time point experiments, cells were grown in 5 mL LB broth in 18 mm test tubes at 37°C in a roller drum and harvested after 4 hours. To determine CtpA substrate stability, saturated cultures were diluted into 25 mL LB broth in a 125 mL flask so that the OD_600_ was 0.05 and grown at 37°C with shaking at 225 rpm until the OD_600_ was ~0.3 (exponential phase) or 3.0 (stationary phase). Protein synthesis was inhibited by adding spectinomycin at 3 mg/mL. Cells were collected by centrifugation at specific time intervals and analyzed by immunoblotting as described below. Band intensities were quantified using ImageJ 1.46r software (National Institutes of Health, Bethesda, Maryland, USA).

### Immunoblotting

Cells were resuspended in 1 × Laemmli loading buffer and boiled for 15 minutes. ∆*ctpA* cell extracts were loaded with 1/5th volume of that of the *ctpA*^+^ extracts. Samples were separated by SDS-PAGE and transferred onto a nitrocellulose membrane by semi-dry electroblotting. The nitrocellulose was blocked in 5% (wt/vol) skimmed milk in phosphate-buffered saline containing 0.1% (vol/vol) Tween 20 (PBST) for 1 h and then incubated overnight in a polyclonal antiserum diluted in PBST, 1:20,000 for α-PA1048, or 1:10,000 for α-CtpA, α-LbcA, and α-PA1198 at 4°C. The membrane was washed with PBST and then incubated in goat anti-rabbit IgG horseradish peroxidase (Sigma, 1:20,000) for 1 h at room temperature. For chemiluminescent detection, the membrane was incubated in ECL Prime detection substrate (Pierce) for 5 min.

### Transposon mutagenesis screen

Φ(PA3923*p-aadA1*) operon fusion strain AJDP1798 was mutagenized with the mariner-based transposon delivery plasmid pBT20 delivered by conjugation from *E. coli*. Transposon insertion mutants were isolated on VBM agar supplemented with gentamicin (75 µg/mL) and tetracycline (75 µg/mL). A bank of approximately 100,000 unique transposon mutants was used for the screen. To identify mutants with increased Φ(PA3923*p-aadA1*) expression, mutant pools were grown in 96-well microtiter plates containing 200 µL of LB broth supplemented with 75 µg/mL tetracycline, 8 mg/mL spectinomycin, and 4 mg/mL streptomycin for 48 hours at ~22°C. Single colonies were then isolated on LB agar and tested for increased antibiotic resistance by their ability to grow on LB agar containing 8 mg/mL spectinomycin and 4 mg/mL streptomycin. These mutants were screened by immunoblot analysis to identify those with an increased steady-state level of CtpA substrate PA1048 compared to the parental strain. Transposon insertion sites were determined by arbitrary PCR and DNA sequencing (55). The insertion upstream of *yohD* (PA5244) was backcrossed into strain PAK by electroporation as described (56) and verified by colony PCR.
